# Linear Growth Impairment in Patients With Pediatric Inflammatory Bowel Disease

**DOI:** 10.7759/cureus.26562

**Published:** 2022-07-04

**Authors:** Hasan M Isa, Masooma S Mohamed, Fawzeya A Alahmed, Afaf M Mohamed

**Affiliations:** 1 Department of Pediatrics, Arabian Gulf University, Manama, BHR; 2 Department of Pediatrics, Salmaniya Medical Complex, Manama, BHR; 3 Department of Public Health, Ministry of Health, Manama, BHR

**Keywords:** bahrain, risk factors, linear growth impairment, children, inflammatory bowel disease

## Abstract

Introduction

Linear growth impairment (LGI) is one of the complications of pediatric inflammatory bowel disease (IBD). This study aimed to assess the linear growth of patients with pediatric IBD and to detect the frequency and the possible risk factors of LGI.

Methods

A retrospective cross-sectional review of medical records of patients with pediatric IBD was conducted in the pediatrics department, Salmaniya Medical Complex, Bahrain, from 1984 to 2019. Demographic and anthropometric data were gathered. World Health Organization (WHO) standards and references were used to define LGI. According to WHO, stunting and severe stunting were defined as length/height for age of <-2 standard deviations and <-3 standard deviations from age and sex-specific reference means, respectively. To determine the possible risk factors for LGI, stunted patients were compared with normal height patients in regard to demographic data, clinical presentations, and treatment used.

Results

Out of 130 patients with pediatric IBD, 88 (67.7%) had anthropometric data available. Fifty-five (62.5%) were males. Forty-seven (53.4%) had Crohn’s disease and 41 (46.6%) had ulcerative colitis. The mean age at presentation was 10.7±3.8 years. The median age at the time of growth measurement was 14.2 (interquartile range=12.1-24.4) years. Fifteen (17%) patients were stunted, and seven (46.7%) of those stunted patients were severely stunted. Weight at presentation was lower in stunted patients (21.6±5.9 kilograms) compared to normal height patients (31±13.4 kilogram) (p=0.048). Sex, delivery type, birth weight, height at presentation, age at presentation, age at growth measurements, IBD type, disease duration, presence of extraintestinal manifestations, and prednisolone and biologic therapy use were not significant factors of stunting.

Conclusion

Patients with pediatric IBD have a high prevalence of LGI compared to the general population. Low weight at disease presentation is the only significant risk factor for LGI. This might indicate that IBD as a disease by itself is having the main negative impact on linear growth.

## Introduction

Inflammatory bowel disease (IBD) is a chronic, relapsing disease [1-2]. IBD is divided into three diseases: Crohn’s disease (CD), ulcerative colitis (UC), and indeterminate colitis [1-4]. It may present at any age, including infancy [1,5]. Being a lifelong disease, patients who present early in life will suffer for a longer time and have more complications [1,5]. Most patients with IBD presented around the age of pubertal growth spurt [6]. Linear growth impairment (LGI) is one of the complications of childhood IBD, and it is considered a significant aspect of the disease [3-4,7-10]. LGI has different synonyms. In preschool children, it is called growth failure, short stature, or stunting while it is called short stature in older children and adolescents [3]. LGI can have a substantial effect on patients’ quality of life and future health and well-being [7].

The exact mechanism of LGI in patients with IBD is not fully understood [6]. Several factors might lead to an LGI in patients with IBD [2-3,9,11-14]. Nutritional, inflammatory, immunological, endocrine, and genetic factors can disturb the growth of patients with IBD and can have a significant impact on the start and progress of puberty [6,11-12,14]. Detection of LGI clinical risk factors might enable earlier intervention and restoration of linear growth [10,15].

The goals of pediatric IBD treatment are to achieve remission, control the disease, and stimulate linear growth to reach the targeted height [6,8,11]. Identification of LGI via a proper assessment is crucial for planning appropriate treatment that can reverse LGI [2-3,6,16]. Initial and continuous growth assessment may greatly improve patients’ health outcomes [7,16]. Despite the availability of several treatment modalities, such as anti-inflammatory, immunosuppressive, and biologic therapies, LGI is still encountered in this group of patients [6,12].

In developing countries, data about the linear growth status of patients with pediatric IBD are limited [3]. Up to date, there are no published studies about LGI in patients with pediatric IBD from the Kingdom of Bahrain. This study aims to assess the linear growth status of patients with pediatric IBD in Bahrain and to detect the frequency and possible risk factors of LGI.

## Materials and methods

In this retrospective cross-sectional study, a review of the paper-based and electronic medical records of patients diagnosed with pediatric IBD in the pediatrics department, Salmaniya Medical Complex (SMC), Bahrain, between January 1984 and January 2019, was conducted. SMC is the main hospital in Bahrain where all patients with pediatric IBD are referred for diagnosis, treatment, and follow-up. All patients who had available anthropometric parameters related to linear growth were included in the study. The diagnosis of IBD was confirmed based on the published criteria [17-18].

Data collection

Data about sex, nationality, type of IBD, age at presentation, disease duration, age at the time of anthropometric measurements, along with medical treatments used, were gathered. Anthropometric parameters at birth, at the initial presentation, and during the last outpatient visit or during the last hospital admission were collected. Height was measured using a Seca digital column scale with a stadiometer (Hamburg, Germany).

The latest growth parameters were calculated using the World Health Organization (WHO) “WHO AnthroPlus” anthropometric software program version 3.2.2 (WHO, Geneva, Switzerland, 2011). These parameters included weight for age (WA) in kilograms, length for age in centimeters for patients less than three years of age, height for age (HA) in centimeters for patients more than three years of age, height for age percentile (HAP), and height for age z-score (HAZ). The parameters were presented as a standard deviation (SD) from age and sex-specific reference means. WHO child growth standards from two to five years of age and WHO growth references for school-age children and adolescents from five to 19 years of age were used as references for the interpretation of linear growth status [19-20]. Accordingly, LGI (stunting) and severe stunting were defined as length/height for ages of less than -2 SD and less than -3 SD, respectively [19-20].

To determine the possible risk factors for LGI, stunted patients were compared with normal length/height patients regarding sex, type of delivery, birth weight, weight and height at presentation, age at presentation, age at growth measurements, type of IBD, disease duration, presence of extraintestinal manifestations and the use of prednisolone and biologic therapy.

Statistical analysis

Statistical Package for Social Sciences (SPSS) program version 21 (IBM Corp, Armonk, NY) was used to analyze patients' data. Categorical variables related to demographic and linear growth status data were presented as frequencies and percentages. Continuous variables were presented as mean ± SD or median and interquartile range (IQR). The Mann-Whitney U test or the student t-test was used to compare continuous variables of anthropometric data and to analyze the continuous risk factors of LGI. The Fisher’s exact or Pearson chi-square test was used to compare categorical variables and to analyze categorical risk factors of LGI. The Kruskal-Wallis test or one-way analysis of variance (ANOVA) was also used to analyze continuous risk factors. The confidence interval was set at 95%. P-values <0.05 were considered statistically significant.

Ethical clearance

Informed consent was obtained from the participants at presentation. This study was ethically approved by the secondary care medical research subcommittee, Salmaniya Medical Complex, Ministry of Health, Bahrain (IRB number: 76120521), and it was conducted in accordance with the principles of the Helsinki Declaration.

## Results

During the study period, 88 (67.7%) out of 130 patients have been diagnosed with pediatric IBD and had linear growth anthropometric data available. The remaining 42 (32.3%) patients with unavailable anthropometric data were excluded. Demographic data of the included patients are shown in Table 1.

**Table 1:** Demographic data of patients with pediatric inflammatory bowel disease Data are presented as number (%) or median (IQR). *Data about IBD medications used was available for 81 patients and each patient might require more than one medication. ^†^two patients received somatotropin alone, one patient received somatotropin and testosterone. IBD, inflammatory bowel disease; IQR, interquartile range

Demographic data		Patients (n= 88)
Sex	Male	55 (62.5)
	Female	33 (37.5)
Type of IBD	Crohn’s disease	47 (53.4)
	Ulcerative colitis	41 (46.6)
Age at presentation (y)	0-4.9	8.0 (9.1)
	5-9.9	27 (30.7)
	10-14.9	43 (48.9)
	15-18	10 (11.4)
Disease duration after diagnosis (y), median (IQR)		4.3 (0.4-10.9)
Age at the time of linear growth measurement (y)	0-4.9	1.0 (1.1)
	5-9.9	9.0 (10.2)
	10-14.9	37 (42)
	15-18	4.0 (4.5)
	>18	37 (42)
IBD medications used, (Patients' n=81)*	Azathioprine	61 (75.3)
	Prednisolone	58 (71.6)
	Mesalazine	53 (65.4)
	Biological therapy	36 (44.4)
	Methotrexate	2.0 (2.5)
	Others^†^	8.0 (9.9)

Fifty-five patients (62.5%) were males, and 33 (37.5%) were females. Seventy-seven (87.5%) were Bahraini nationals, and 11 (12.5%) patients were non-Bahraini (four patients were from India, two from Egypt, Syria, and Pakistan each, and one from Morocco). Forty-seven (53.4%) patients had CD and 41 (46.6%) had UC. The mean age at presentation was 10.7 ± 3.8 years. Eight (9.1%) patients had an early onset disease (below the age of five years). The median age at the time of growth measurement was 14.2 years (IQR=12.1-24.4). The most common clinical presentations were recurrent abdominal pain (39 (76.5%) patients) followed by bloody diarrhea (42 (68.8%) patients). The most frequent medications used were azathioprine (61 (82.4%) patients) and prednisolone (58/76 (76.3%) patients). Biological therapies were used in 36 (44.4%) patients (21 received adalimumab, 10 received infliximab, four received both adalimumab and infliximab, and one received biologic therapy of unspecified type). Exclusive enteral nutrition (EEN) therapy (Ensure) was used in three patients.

Basic anthropometric data of all patients are all shown in Table 2.

**Table 2:** Basic anthropometric data of 88 patients with pediatric inflammatory bowel disease IQR, interquartile range; SD, standard deviation; HAP, height-for-age percentile; HAZ, height-for-age z-score

Anthropometric parameters		Results
Birth weight (kg), median (IQR), (n=39)		3 (2.9-3.4)
Weight at presentation (kg), median (IQR), (n=32)		26.2 (13.4)
Height at presentation (cm), mean ± SD, (n=15)		128.9 ± 14.9
Age at growth measurements (y), median (IQR)		14.2 (12.1-24.4)
Weight-for-age (kg), mean ± SD		51.2 ± 20.3
Height-for-age (cm)	median (IQR)	154 (140-165)
	mean ± SD	151.9 ± 16.6
HAP, median (IQR), (n= 81)		22.7 (6.4-49.9)
HAZ, mean ± SD, (n=51)		-0.88 ± 1.46

Comparison based on the type of IBD showed no significant differences between patients with CD and those with UC in their basic anthropometric parameters (Table 3).

**Table 3:** Comparison of anthropometric data of patients with Crohn’s disease and those with ulcerative colitis CD, Crohn’s disease; UC, ulcerative colitis; CI, confidence interval; hAP, height-for-age percentile; HAZ, height-for-age z-score Data are presented as mean ± SD. P-values are resulted from the ^a ^Mann-Whitney U test or the ^b ^student t-test.

Anthropometric parameters	n	CD patients (n=47)	n	UC patients (n=41)	P-value (95% CI)
Birth weight (kg)	20	3.0 ± 0.3	19	3.2 ± 0.4	0.449^a^
Weight at presentation (kg)	15	28 ± 9.4	17	29.3 ± 15.1	0.766^a^
Height at presentation (cm)	6	136.5 ± 7.7	9	123.8 ± 16.7	0.07^b^ (-1.2-26.7)
Age at growth measurements (y)	47	17.3 ± 6.6	41	19.5 ± 10.8	0.913^a^
Weight-for-age (kg)	47	50.8 ± 19	41	51.7 ± 22	0.843^b^ (-9.65-7.89)
Height-for-age (cm)	47	155.2 ± 13.9	41	148.2 ± 18.7	0.061^a^
HAP	43	29 ± 26	38	31 ± 29	0.876^a^
HAZ	29	-0.95 ± 1.4	22	-0.8 ± 1.54	0.723^b^ (-1.0-0.701)

**Figure 1:**
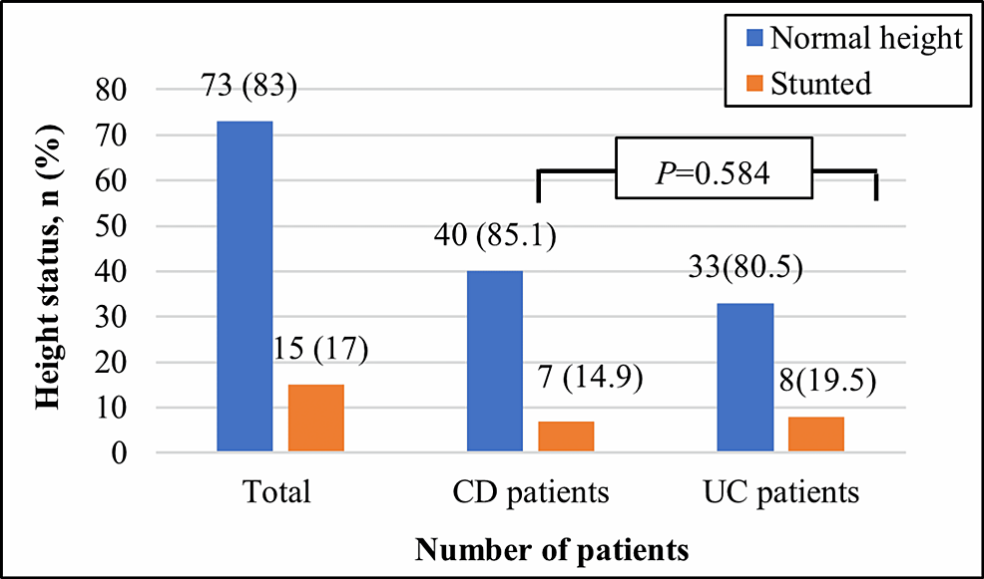
Linear growth of patients with pediatric inflammatory bowel disease CD, Crohn’s disease; UC, ulcerative colitis. Data presented as number (%). P-values are resulted from the^ a ^Fisher’s exact test.

Results of linear growth assessments are shown in Figure 1.

Seventy-three (83%) patients had normal height measurements while 15 (17%) patients were stunted (HA <-2 SD); seven (46%.7) of them were severely stunted (HA <-3SD). There were no significant differences between patients with CD and those with UC in the linear growth assessment.

Univariate analysis of the possible risk factors of LGI is shown in Table 4.

**Table 4:** Univariate analysis of linear growth impairment risk factors in patients with pediatric inflammatory bowel disease IBD, inflammatory bowel disease; SD, standard deviation; CI, confidence interval Data are presented as number (%) or mean ± SD. P-values are resulted from the ^a ^Fisher’s exact test, ^b ^Mann-Whitney U test, or ^c ^student t-test.

Variables			Linear growth of 88 patients with IBD		P-value
			Normal = 73 (83%)	Stunted = 15 (17%)	
Sex		Male	48 (65.8)	7.0 (46.7)	0.241^a^
		Female	25 (34.2)	8 (53.3)	
Type of delivery		Vaginal	20 (90.9)	7.0 (100)	>0.999^a^
		Caesarean	2.0 (9.1)	0.0 (0)	
Birth weight (kg), mean ± SD			3.2 ± 0.4	2.9 ± 0.4	0.072^b^
Weight at presentation (kg), mean ± SD			31 ± 13.4	21.6 ± 5.9	0.048^b^
Height at presentation (cm), mean ± SD			129.8 ± 16.6	126.3 ± 10.4	0.395^b^
Age at presentation (y), mean ± SD			10.9 ± 3.7	9.6 ± 3.9	0.201^c^ (CI -0.74-3.5)
Age groups at presentation	<10 years		28 (38.4)	7.0 (46.7)	0.574^a^
	10-18 years		45 (61.6)	8.0 (53.3)	
Age at growth measurements (y), mean ± SD			18.2 ± 8.6	19.1 ± 10.3	0.991^b^
Age at growth measurements	<20 years		45 (61.6)	11 (73.3)	0.558^a^
	≥20 years		28 (38.4)	4.0 (26.7)	
Type of IBD	Crohn’s disease		40 (54.8)	7.0 (46.7)	0.584^a^
	Ulcerative colitis		33 (45.2)	8.0 (53.3)	
Disease duration before diagnosis (mo), mean ± SD			1.1 ± 1.7	1.56 ± 2.1	0.488^b^
Disease duration after diagnosis (y), mean ± SD			6.4 ± 7.3	9.6 ± 9.5	0.252^b^
Extraintestinal manifestation at diagnosis			12 (40)	3.0 (42.9)	>0.999^a^
Prednisolone use			48 (77.4)	10 (71.4)	0.730^a^
Biological therapy use			28 (45.2)	8.0 (57.1)	0.556^a^

Weight at the time of disease presentation was significantly lower in stunted patients compared to normal height patients with a mean weight of 21.6 ± 5.9 versus 31 ± 13.4 kilograms, respectively (p=0.048). Factors like sex, type of delivery, birth weight, height at presentation, age at presentation, age at growth measurements, type of IBD, disease duration, and presence of extraintestinal manifestations, and the use of prednisolone and biologic therapy were not found to be significant risk factors of stunting.

## Discussion

This study showed the prevalence rate of LGI among patients with pediatric IBD to be 17%, which is much higher than that of the general population [21-22]. In Bahrain, the prevalence of LGI in children up to five years of age and children 10 to 12 years of age was 5.8% and 4.2%, respectively [21-22]. However, El Mouzan et al., Sentongo et al., and Motil et al. studies reported a higher percentage of stunting in patients with pediatric IBD: 26%, 25%, and 23%, respectively [3,14-15]. Nonetheless, the prevalence of stunting in our study is still higher than that reported by Aurangzeb et al. and Song et al. studies where only 3.6% and 4% of their pediatric patients with IBD were stunted; respectively (Table 5) [7,16].

**Table 5:** Linear growth impairment in patients with inflammatory bowel disease in neighboring countries and worldwide IBD, Inflammatory bowel disease; UC, Ulcerative colitis; CD, Crohn’s disease; USA, United States of America the present study. ^† ^the number of stunted patients was not reported in the published article and was calculated based on the reported percentages.

Country	Author/Year	Study period	n	IBD Type	Age (Year)	Stunted n (%)
Bahrain⃰	Isa et al. 2022	1987-2019	88	Both	5-19	15 (17)
Saudi Arabia	El Mouzan et al. 2016 [3]	2003-2012	374	Both	0.33-16	97 (26)
Australia	Aurangzeb et al. 2011 [7]	2011	28	Both	0.5-16	1.0 (3.6)
USA	Sentongo et al. 2000 [14]	1999	132	CD	5-25	33 (25)^†^
USA	Motil et al.1993 [15]	1979-1983	69	Both	12.6±3.3	16 (23)^†^
Korea	Song et al. 2014 [16]	1996-2011	71	CD	<18	3.0 (4.0)

This difference in the prevalence of LGI may be attributed to the fact that children in Bahrain are slightly taller in recent years compared to their counterparts 10 to 15 years ago [23]. The changes in children’s growth patterns might be due to the improving living condition in Bahrain [23]. Genetic factors might also have a control on the patients’ height [23]. Furthermore, the differences in LGI between our patients and those from other countries can be explained by the differences in the way of assessing linear growth. Local anthropometric data are not available in Bahrain. Accordingly, WHO definitions and references were used for LGI assessment and to compare our results with other populations. Using WHO references makes the comparison between different studies easier [3]. However, this might overestimate the prevalence of short stature [24]. Additionally, some studies used their national growth charts or the Centers for Disease Control and Prevention (CDC) references, which makes the comparison harder to achieve [15,24-25].

In this study, the univariate analysis of the possible risk factors of stunting showed that weight at presentation was the only significant risk factor (p=0.048). Stunted patients had lower weight at presentation compared to those with normal height. This finding was not reported by other studies. Linear growth is regulated by multiple factors including food intake and hormones [26]. Malnutrition and obesity can lead to linear growth changes [26].

Our study did not show a significant effect of sex on linear growth. This is similar to El Mouzan et al.'s study [3]. However, a case-control study published by Sentongo et al. showed that LGI was significantly more common in males (26%) than females (23%) in 132 patients with CD [14]. The authors attributed their findings to the greater concern about height among males, which subsequently leads to earlier referral and diagnosis of CD in males [14]. Nevertheless, Song et al.'s study showed that female sex was a significant risk factor for lower height for age z scores [16].

The present study could not demonstrate a significant effect of age (neither at presentation nor at the time of the study) on linear growth. Likewise, Sentongo et al.'s study revealed no effect of pubertal age on the prevalence of LGI in patients with CD [14]. However, El Mouzan et al.'s study showed a significantly higher prevalence of LGI in young children with CD and in older children with UC [3]. Patients with early-onset IBD (before puberty) are often short, as these patients will have a considerably slower growth rate and lower final height compared to their parental target [12]. Yet, a prospective study by Motil et al. showed that the height deficits were equally prevalent at the time of study regardless of the stage of pubertal development [15]. Nevertheless, on long-term follow-up, the prevalence of LGI in pubertal children was higher than that in prepubertal children (40% versus 16%, P<0.01) [15].

This study did not show a significant effect of the type of IBD on the prevalence of LGI. This is similar to El Mouzan et al.'s study [3]. However, Motil et al.'s study found that patients with CD were twice likely to have LGI compared to those with UC [15]. Also, Sentongo et al.'s study showed a low height for age score in CD patients [14]. This might be explained by the wide spread and the longer disease duration before diagnosis in CD compared to UC [6,9,12]. Moreover, CD is very difficult to manage [11]. Nevertheless, UC has less systematic inflammation and consequently less effect on bone, appetite, and nutrition [6].

Our study did not show a significant effect of disease duration on linear growth. Similarly, Sentongo et al. and Motil et al.'s studies showed no effect of disease duration on LGI in patients with CD [14-15].

Unlike our study, Song et al.'s study showed that the presence of extraintestinal manifestations in patients with CD at diagnosis was a significant risk factor for lower height for age z scores [16]. This might be related to high levels of anorexigenic pro-inflammatory cytokines that can affect linear growth [16].

The effect of steroid therapy on the development of LGI in children with IBD is controversial [15,27]. Although glucocorticoid use can suppress bone growth, early LGI at the time of IBD diagnosis indicates that it is a complication of the disease itself [6,12,26]. LGI is related to the inflammatory process and not just an adverse effect of steroid use [11,15,26]. Our study did not show a significant effect of prednisolone use on linear growth. Sentongo et al. and Motil et al.'s studies also revealed no effect of lifetime steroids on linear growth [14-15]. Yet, high doses of steroids over long periods of time are inclined to increase the risk of LGI in patients with CD [11,15]. Accordingly, steroid use should be limited to induce remission and prolonged use should be avoided when possible [6,11,27]. Alternatively, enteral nutrition should be considered to improve linear growth regardless of inflammation status and the use of other medical therapies [27].

The effect of biologics on linear growth is promising, and they have a positive impact on growth [8-9]. Our study did not show a significant effect of the use of biological therapy. However, their role remains controversial and multifactorial as most of the patients who require biologic therapy already have a severe disease at presentation [6,8].

This study was limited by its retrospective nature. Accordingly, missing some relevant data is expected. Moreover, being cross-sectional, this study could not report on the long-term impact of IBD on growth. In addition, disease activity was not assessed in this study. Disease severity is an imperative predictor of LGI [11-12,14]. The Motil et al. study reported a significant negative association between linear growth and disease activity in children with IBD [15]. On the contrary, Sentongo et al.'s study revealed no effect of disease activity on LGI in patients with CD [14]. Furthermore, in this study, linear growth assessment depended on measuring the patient’s height for age. Height for age can express linear growth in children [26]. Nevertheless, HA alone might be misleading, as it is affected by parental height and pubertal status. It might also underestimate the linear growth of some children, as patients with relatively normal heights might have grown little over time [9,26]. HA is considered a crude measure to assess linear growth and can only give little information about its improvement [8]. Parent-specific adjusted height is a more powerful method of assessing LGI, as it can predict linear growth genetic potential [14]. It is better to assess linear growth using height velocity [7,9]. Moreover, this can be calculated as the difference between serial measurements of height in a given time interval (minimum of six months) and is usually presented in centimeters per year [9,26]. The height velocity z score of age and sex is the earliest and most sensitive parameter for the detection of LGI [9].

Finally, an assessment of pubertal status using Tanner staging was not performed on our patients. Yet, it should be integrated into the growth assessment [9]. Despite these limitations, the findings of this study are important being the first study to tackle LGI of patients with pediatric IBD in Bahrain and can form a foundation for future studies.

## Conclusions

This study showed a high prevalence of linear growth impairment in patients with pediatric IBD as compared to the general population. Low weight at disease presentation was the only significant risk factor for LGI. This might indicate that IBD as a disease by itself is having a major negative impact on linear growth. More studies on the impact of pediatric IBD on height velocity, puberty final adult height, as well as body composition, are needed. Further studies on the effect of various therapeutic options that might reverse stunting are of particular importance.
